# On Artificial Dentures

**Published:** 1859-01

**Authors:** J. Allen


### ARTICLE XV.

### *On Artificial Dentures.* By Dr. J. Allen.

*Gentlemen and Members of the American Dental Convention:*

Another year has passed since last we met, which adds one more to the preceding thousands through which we trace the history of our profession, a retrospective view of which might be dwelt upon with great interest, would time permit, but as our march is onward, we propose to call your attention at once to the present and prospective future. Owing to the vast extent and variety of topics to which dental science pertains, we shall, upon this occasion, touch only upon one branch which will be that of artificial dentures, and leave the other branches to those who will present them in their strongest light.

Of this I am well assured, when I look at the bright horizon of our profession, and see that many of the stars with which it is decked, are here in council assembled. From the east, from the west, from the north, and from the south, there are able representatives here, who have brought with them the rich fruits of experience, ready to co-operate with each other, in order to promote the best interests pertaining to dental science. No political discussions—no local interests—no sectarian differences, or professional jealousies, are permitted to mar the harmony of our deliberations. We come with that national strength and bond of union which characterizes American institutions. We come as humble recipients on the one hand, and cheerful dispensers on the other, of those professional trophies which reflect as beacon lights from the great store-house of knowledge, those brilliant rays which illumine the mind and point us onward in our course. But, although as Americans, we have reason to indulge in a degree of self-complacency for the acknowledged merit accorded to our profession in this country, yet it might be admitted there are many radical defects in our practice which ought to be corrected. It will, therefore, be our present purpose to refer to some of the most prominent of these in connection with artificial teeth, and endeavor, as far as in our power, to point out the remedies, as based upon personal experience.

In the construction of artificial dentures, there are four very important points to be attained, which are too often overlooked, not understood, or duly appreciated. These are, utility in mastication, clear and distinct enunciation, a natural or comely expression, and restoration of the face in cases where it has become sunken.

I shall endeavor to elucidate each of these points in practical detail, as they involve several others worthy of close attention.

The first point for consideration is, Utility in Mastication, which involves adaptation of the plate or base, and a correct position and articulation of the teeth. With the mind's eye, we will now examine a set of teeth which cannot be used for masticating food, as they become readily dislodged in attempting to chew. The cause is owing to a misfitting plate, the defect appears to have been in the impression which was taken with wax, and probably became slightly bent on being removed from the mouth. To remedy this, if wax be used, it should be hardened by cooling before being removed. For this purpose the impression-cups invented by Dr. Harrison, of ——, New York, are the most convenient for chilling the wax, or gutta percha, which is preferable, especially for partial sets.

These cups are made with double plates, and permit a current of cold water to pass around through the cup between the plates, which chills the impression material sufficiently to retain its form on being removed from the mouth. But good plaster of paris, with proper manipulation, is preferable for taking impressions. The impression being properly taken, we will next proceed to notice what we deem the preferable mode of forming dies. It consists in pouring molten metal upon the counter-impression from the mouth, which is composed of equal parts of plaster and sand (with water to mix ;) the sand is used to prevent the plaster from shrinking, by the heat to which it is subsequently subjected. The counter-impression should be thin, and placed in an iron flask or cup, and a mixture of plaster and sand, as above, is poured around it in the flask, which hardens and fixes the model firmly in the iron cup. It should then be thoroughly dried and gradually heated to about two hundred and twenty degrees, it is then removed from the oven of the stove or furnace, and placed in a large pan. A heated iron, red hot, like a large soldering iron, should be placed upright, with the heated point resting upon the centre of the model, and then the melted metal is run in the flask upon the model. On cooling, the only point of shrinkage takes place around the heated iron. The metal should be poured at as low a degree of heat as possible. This metal is composed of equal parts of lead and tin, and is called the base. To facilitate the cooling of the base, a stream of cold water 'may be run into the pan in which the flasks sits, which hardens the metal, after which the heated iron is removed and a little more metal is run into the vacuum it occasioned. This done, the metal is removed from the flask or cup which is made flaring for the purpose, and the plaster and sand model being removed from the metal, a base is thus formed upon which several counter-casts may be run, with which a plate can be struck with more accuracy than by any other method with which I am familiar.

These counter-casts should be made of a compound that will melt at a lower heat than the base, which we form of one part lead, one part tin, and two parts of bismuth. A little sulphate of copper in solution, may be brushed over the surface of the base, before running on the tops, to prevent them from sticking to the base. A rim of iron or putty should be placed upon the base to run the tops in. Putty may also be used to advantage in undercut cases, by filling the undercut in the base on one side, and then running on a top which will be easily removed, then remove the putty from that point, and place it upon the opposite side, and run on another top, and by these means a most perfect fitting plate may be formed. We usually run on three or four of these tops, which will enable the workman to bring up his plate sharp and true at every point.

I would here state that to Dr. Gunning, of New York, we are indebted for this method of taking casts.

We will now examine another full set of teeth. The impressions in this case were properly taken, the dies were rightly formed, and the plates fit the mouth for which they were intended, yet the same difficulty is experienced by the wearer as in the former case ; but it arises from another cause—the teeth are set so far out upon the plates as to cause them to become dislodged from one side; when pressure is borne upon the opposite. This is a common fault, and will defeat a good, practical result, though every other point in the case be right. To remedy this defect, the teeth should be so arranged upon the plate as to have the pressure when the teeth are closed, bear upon the inner rather than the outer margin of the alveolar ridge. This will prevent the plates from becoming dislodged from one side when biting upon the other. Upon this point the dentist should exercise the skill and judgment of the architect who builds his superstructure with special reference to his foundation, taking care that the structure shall not overhang the base which is to sustain the weight or pressure.

We will now examine a full denture made for the Rev. Mr. D——. The plates are well fitted to the mouth ; but his voice, once so audible and clear, is now flat and indistinct ; his enunciation, once so pleasing, has become laborious to himself and painful to his hearers. The defect in this is, his artificial denture is so unnatural in form that the tongue cannot adapt itself to it, so as to produce distinct enunciation. Hence the muffled or hissing sounds in speaking.

In the construction of a musical instrument with reeds or tongues, the most perfect adaptation of the surrounding parts is necessary, in order that each note shall have a round, full, and clear tone ; the slightest defect in this respect throws the instrument out of tune, and discordant notes are then produced. So with the human voice ; in order that the notes and words be clear, full, and melodious, a perfectly natural form should be given to artificial teeth and gums, that they may be properly adapted to the tongue. To this end, the continuous gum, roof, and ruga of the mouth, as practiced by your speaker, is admirably adapted to the accomplishment of this object.

The next set to which we would call your attention is in the mouth of Miss E——. You need not be told they are artificial, for the first glance reveals the fact which is extremely mortifying to her and her friends: for she is by nature and education a lady of taste and refinement. With one exception (her teeth) her features are classic, her manners gentle and engaging. But the ruthless hand of time or disease, has robbed her of one of the brightest ornaments of the face. There is no feature in which there is more expression, or that commands more admiration, than a beautiful set of teeth. But from her they are gone forever, and, like brilliant gems rudely torn from the casket, there is only left the marred relics that contained them. An attempt has been made, as you see, to restore her loss by artificial means, but the artist was not there.

The greatest defect in *these* teeth, is a total want of natural expression. In order to remedy this defect there are several points to be observed, viz. the length, the size, the shade, and the position of the teeth.

The length should depend upon the width of the lips that are to cover them. As a general rule, the front teeth should be long enough to display their points or cutting edges a little below the upper and above the under lip, when in their natural position. The side and back teeth should be of such length as to permit the lips to come together without compression. This precaution, when getting the bite, will give to the face its due proportion of length, and display an agreeable expression of the teeth.

The size should bear a due proportion to the other features of the face.

The shade should harmonize with the complexion of the person for whom they are intended.

Here the skill of the artist is requisite in order to avoid an unnatural contrast that would lead to detection. The dentist who is a true artizan, is not ambitious to have his work bear the impress of artificial teeth, but on the contrary, that they should exhibit that depth of tone, natural form, and truthful expression, which characterize the natural organs. The position of the front and eye teeth gives character in appearance to the mouth. An undue inclination outward, or inward, or oblique, will change the expression just in proportion as they are varied from the original position of the natural teeth. Hence, in many persons their former expression is almost entirely lost, and distortion takes the place of symmetry. This defect in the arrangement of teeth, is a common error in the construction of artificial dentures, especially when done by mere mechanical dentists, whose rude and graceless work exhibits a striking contrast with that of the artizan who carefully studies the tone, position, and expression of every tooth, and restores the harmony which nature had originally stamped upon the features of his patient. A few slight touches of the brush in the hands of a skillful artist, will change the whole expression of his picture. So with the teeth, a slight inclination outward or inward, or variation in length, will change the entire expression of the mouth.

In articulating a set of teeth, there are three important points to be observed. First. The front lower incisors should not infringe upon the front teeth above, as this would tend to dislodge the upper set. Second. The pressure, when the teeth are closed, should come upon the *inner* cusps of the molar and bicuspid teeth rather than upon the outer. This precaution will, in many instances, prevent their displacement from one side, when chewing upon the opposite. Third. The bicuspids and molar teeth of the upper and lower set, when closed, should interlock with each other like the natural teeth. This will render the process of mastication much more easy than if the teeth strike perpendicular upon each other.

Another important point in the construction of artificial dentures, is the restoration of the face in cases where it has become sunken. There are four points of the face which the mere insertion of artificial teeth will not always restore, viz. one upon each side beneath the malar or cheek bone, and one upon each side of the base of the nose in a line towards the front portion of the malar bone.

The muscles situated upon the sides of the face (as you are aware) and which rest upon the molars, are the zygomaticus major masseter and buccinator; the loss of the above teeth in many persons, cause these to fall in. The principal muscles which form the front portion of the face and lips are the zygomaticus minor, levator labii superioris alæque nasi and orbicularis oris. These rest upon the front, eye, and bicuspid teeth, when these teeth are lost, the muscles fall in. This, together with the absorption of the alveolar processes, changes the form and expression of the mouth.

The insertion of the front teeth will, in a great measure, bring out the lips; but there are *two muscles* on each side of the nose in the front portion of the face which cannot, in many persons, be thus restored to their original position. One is the zygomaticus minor, which arises from the front part of the malar bone, and is inserted into the upper lip, above the angle of the mouth. The other is the levator labii superioris alæque nasi, which arises from the nasal process, and from the edge of the orbit, above the infra orbitar foramen, and is inserted into the ala nasi or wing of the nose and upper lip.

In order to restore these sunken portions of the face, prominent attachments should be formed upon the dentures of such size and shape as to raise these muscles to their original fullness, thus rejuvenating the sunken cheek.

Here again the artistic skill of the dentist is brought into requisition. He should study the face of his patient as the artist studies his picture; for here he displays his genius. not upon canvas, but upon the living features of the face. And of how much importance is the living picture that reflects even the passions and emotions of the heart, than the lifeless form upon canvas?

We will now examine a full set of teeth which combines all the requirements of an artificial denture. The plates are well adapted to the mouth of the wearer. The teeth are properly arranged upon the plates, and display a comely and pleasant expression. The tone of the teeth are truthful in appearance, and are garnished with a continuous gum-roof and ruga of the mouth, which will vie with those produced by nature. The inside form is well adapted to the tongue. The sunken or dilapidated face is rejuvenated, and the patient is now ready to exclaim—"Richard is himself again!"

I deem it proper here to urge upon our brethren the great importance of bringing into requisition a much higher order of talent in the artificial branch of our profession than has heretofore been employed by a large number of dentists whose ambition prompts them to do the cheapest, not the best work. This low ambition always has a downward rather than an upward tendency, and will place its votaries where they are sure to be found, upon the lower platform of their profession. These penny wise and pound foolish practitioners are dead weights upon our fraternity, and do much to retard the progress of improvement. *They* will not tax *their genius*, for they are not remunerated. They will not hazard expenditure to ascend the hill of science, for it will not pay. They will not incur much expense in doing work, for their prices will not justify it. But the aspirant for the highest crest upon the mountain top, ignores such sordid considerations; he struggles onward and upward. If he meets with obstacles, he surmounts them. If discouragement and difficulty stare him in the face, he frowns them down, keeping his eye steadily upon the goal where he had placed his mark. He believes that what man can do, he can. Nay, more, he believes that what ought to be done, must be done, and he is going to do it. His restless zeal will not permit him to wait for some predecessor to do the pioneering; he does it himself, let the cost be what it may, or the labor never so much. Labor is the secret of success. [*Dent. Register.*

